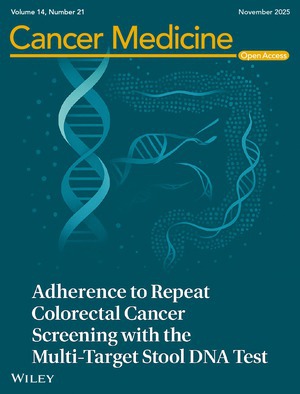# Cover Image

**DOI:** 10.1002/cam4.71381

**Published:** 2025-11-19

**Authors:** Mallik Greene, Joseph Anderson, Joseph LeMaster, Jeffrey Arroyo, Jorge Zapatier, Jemel Bingham, Raja Kakuturu, Jordan J. Karlitz, Quang Le

## Abstract

The cover image is based on the Research Article *Real‐World Adherence to Repeat Colorectal Cancer Screening With the Multi‐Target Stool DNA Test in a Large, Insured, and Average‐Risk Population* by Mallik Greene et al., https://doi.org/10.1002/cam4.71314.